# Assisted implant stabilization using a modified microplate fixation technique

**DOI:** 10.1016/j.jobcr.2025.06.019

**Published:** 2025-06-30

**Authors:** Mohamed Hamdy Helal, Ahmed Noaman Ali, Eman Abd El Raouf Tawfik, Mostafa Mahmoud Youssef Mohamed, Moustafa Nabil Aboushelib

**Affiliations:** a Oral Medicine, Periodontology, Oral Diagnosis and Radiology Department, Faculty of Dentistry, Tanta University, Egypt; b Department of Basic Dental Sciences, Faculty of Dentistry, Zarqa University, Zarqa, Jordan; c Oral Pathology Department, Faculty of Dentistry, Tanta University, Egypt; d Dental Biomaterials Department, Faculty of Dentistry, Tanta University, Egypt; e Department of Clinical Dental Sciences, Faculty of Dentistry, Zarqa University, Jordan; f Dental Biomaterials Department, Faculty of Dentistry, Alexandria University, Egypt

**Keywords:** Animal study, Assisted implant stabilization, Microplate fixation technique, Osseointegration

## Abstract

**Objectives:**

In many clinical cases, the surgical defect around the osteotomy does not grant sufficient stability which may compromise the inserted dental implant. Using a fixation microplate may solve this issue by improving the stability of the inserted implant. This study aimed to evaluate assisted implant stability using microplate fixation in compromised bone defects.

**Methods:**

Two osteotomy defects (6 mm and 4 mm in diameter) were made in the femur of a dog model. Dental implants (4 mm in diameter and 10 mm in length) were placed in the two defects. Microplates were used to enhance the initial stability of the compromised implant placed in the larger defect size without the need for the additional use of a bone graft. A reverse torque test and histomorphometric analysis were performed after eight weeks to assess bone implant contact (n = 48).

**Results:**

Both plate and control implants had almost identical bone-to-implant contact ratios, 82.55 ± 0.61 and 82.86 ± 0.69, respectively. The two tested implants had no statistically significant difference in bone implant contact (t = 1.155, *P* = .260) nor reverse torque test (t = 1.408, *P* = .173).

**Conclusions:**

Assisted implant stabilization improved osteointegration of implants suffering from poor initial stability without the need to resort to bone graft or other complicated techniques.

## 1 Introduction

The primary and secondary stability of endo-osseous implants play a significant role for their optimal osseointegration and long-term clinical success 1. Primary implant stability is determined by the implant direct mechanical contact with the neighboring bone, whereas secondary implant stability is a controlled by direct adaptation of newly created bone on the surface on the inserted implant.^1^ Secondary stability is inextricably linked to successful functional loading of the final restoration.^2^ The extent of implant stability is determined by the state and quality of the surrounding tissues.^3^, ^4^, ^5^ Primary stability is influenced by a variety of clinical parameters, including bone amount and quality, implant design, and surgical technique.^6^

The initial inflammatory stage at the osteotomy site begins with the production of a blood clot, followed by a series of biological healing processes ending in direct bone deposition on the implant surface.^4^ To prevent dental implant movement in the early phases, primary stability is required to prevent fibrous encapsulation which can lead to implant failure.^7^^,^^8^ However, advanced ridge resorption, massive cyst enucleation,^9^^,^^10^ tumor resections,^11^ and a lack of appropriate bone dimensions for the support of oral implants may complicate the rehabilitation plan and affect the treatment modalities. In such compromised conditions, ridge augmentation is recommended to improve bone quality around the compromised implant.^12^

As a result, research has concentrated on the development of innovative procedures for ridge augmentation prior to the insertion of dental implants.^13^^,^^14^ These extra complicated procedures increase the expenses of treatment and dramatically extend the healing time, in addition to their known risks and expected post-operative complications.^15^ Nevertheless, insertion of a dental implant during grafting procedure may shorten treatment time but that will be on expense of gaining sufficient primary stability.

Surgical fixation microplates could be used to engage the connection part of a dental implant utilizing the provided cover screw. Modified fixation microplates could grant assisted stability to implants inserted in compromised sites. The aim of this study was to enhance primary stability of dental implants inserted in large defects using a modified fixation screw technique. The proposed hypothesis was that assisted implant stability would enhance osteointegration without the need for a prior grafting procedure.

## 2 Materials and Methods

### 2.1 Study design

The study protocol was approved by ethics committee of Tanta University's specifying conditions and constraints for conducting and publishing studies involving animal models (No. R-OMPDR-6-23-1) following the Animal Research: Reporting of In Vivo Experiments (ARRIVE) guideline.^16^

### 2.2 Sample size calculation

The sample size was calculated using G-Power software version 3.1.9.2^17^ to detect difference in torque testing. Based on Cochran et al.,^18^ results, and adopting a power of 80 % (β = 0.20) to detect a standardized effect size in torque testing (primary outcome) of 0.515, and level of significance 5 % (α error accepted = 0.05), the minimum required sample size was found to be 12 specimens per group (number of groups = 4) (Total sample size = 48 specimens).^19^^,^^20^

### 2.3 Experimental animals and their housing and husbandry

The animal-house veterinarian evaluated 12 mature male 2-year-old beagle dogs weighing between 10 and 12 kg to rule out disease and ensure that they were provided with a balanced diet of milk, broth, and meat during the study period. All animals were kept in individual stainless-steel cages with direct access to water, proper ventilation, and a 12-h light/dark cycles.

Each animal received two implants in the same femur. Femur site allowed easy and controlled access allowing precise insertion of implants and microplates. It also facilitated post-surgery care and hygiene. On the contrary, the oral cavity would require an additional extraction procedure thus extending healing time and is more prone to infection.

### 2.4 Surgical procedure

**Fig. 1:**
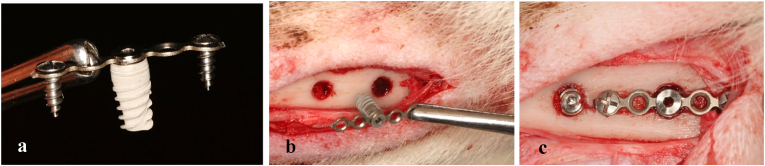
Digital photograph showing the mechanism of assisted microplate fixation technique (a). Digital photograph showing osteotomy sites before securing microplate. 6 mm defect size on the right (b). Digital photograph showing osteotomy sites after securing microplate using mini screws. Compromised implant on the right not touching any bone wall (2 mm gap size) (c).

In this study, four osteotomy sites were prepared in the femurs of 12 adult male dogs (4 and 6 mm in diameter). A standardized implant (4 mm in diameter and 10 mm in length) was inserted at each osteotomy site. A fixation bar was used to stabilize the implant with compromised primary stability, Fig. 1-a. The thickness of bone in the selected areas allowed full coverage of the entire implant length.

To prevent postoperative infection, a prophylactic antibiotic (ampicillin, 25 mg/kg body weight) was administered to each animal immediately prior to the procedure. An expert surgeon (Y. H.) performed all the surgeries in a sterile environment in a veterinary operating room. The dogs were given a subcutaneous injection of atropine (0.05 mg/kg; Kwang Myung Pharmaceutical) and after 10 min of premedication, anesthesia was induced by injecting a mixture of 2 mg/kg Xylazine (Xyla-Ject; Adwia Pharmaceuticals) and 5.5 mg/kg ketamine hydrochloride (KETAMAX-50; Troikaa Pharma) into the cephalic vein of the forelimb and maintaining it with inhalation anesthesia.

**Fig. 2:**
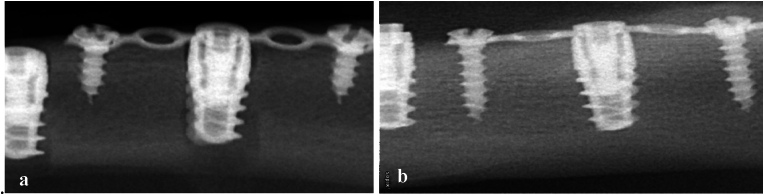
Digital x-ray images (scale bar 10 mm) showing dental implants on day of insertion. Notice larger osteotomy site appearing around compromised implant on the right (a). Digital x-ray image showing the compromised dental implant after 8 weeks. Notice complete healing of the large osteotomy site (b).

The skin over the medial side of the femoral bone was incised and reflected, and the superficial fasciae, muscle tissue, and deep fasciae were incised bluntly. The periosteum was then incised to reveal the femoral shaft, which was ready to receive the dental implants. One sequential osteotomy was started with a pilot drill, followed by 2-, 3.5-, and 4-mm drills to a depth of 10 mm. A second osteotomy, 2 cm apart, was performed with a diameter of 6 mm. Dental implants (B&B DURA-VIT -EV, Italy) of the required size (4 mm diameter and 10 mm length) were inserted into the two prepared osteotomy sites. A micro plate fixation bar (1.2 System Micro, 0.5 mm thickness, 18 hole Plates BioMaterials, Korea) was secured over the compromised implant using the cover screw of the manufacturer and two short fixation pins, Fig. 1-b&-c. Deep muscles and fascia were sutured using absorbable suture (4:0 cat gut suture, Trugut, India) followed by skin using non-absorbable sutures (1:0 black silk, Assut, Egypt), and the surgical site was covered with soft cloths to prevent infection, Digital radiographs were taken immediately after surgery and at each subsequent follow-up, Fig. 2.

### 2.5 Reverse torque test (RTT)

After eight weeks of healing, the implants were uncovered and connected to a surgical handpiece connected to a calibrated step motor using a motor-driven hex tool. The motor was activated in an anticlockwise direction to reach a maximum torque of 50 N cm over 30 s. The test was stopped once the implant started to rotate and the rotating torque was recorded.

### 2.6 Histomorphometry

The animals were sacrificed with an overdose of thiopental sodium after eight weeks, and their femur blocks were dissected. The blocks were fixed for one week in 4 % buffered formaldehyde. The specimens were then dehydrated in increasing ethanol concentrations (50, 70, 90, and 100 %) using a dehydration machine (ASP 300S, Leica Biosystems) with agitation and vacuum. The blocks were embedded in chemically polymerized methyl methacrylate resin, cut in a coronal-apical plane using a precision-cutting machine (Metkon Micracut150 precision cutter), and then ground and polished with 800-grit silicon carbide paper. After staining, (Stevenes blue and Van Gieson picrofuchsin), the sections were examined using a light stereomicroscope (BX61; Olympus Corp) equipped with a high-resolution digital camera (E330; Olympus Corp). Bone implant contact (BIC) was calculated as the amount of new bone in direct contact with the implant surface and as a percentage of the implant perimeter calculated from the most central section.

Data were collected and entered into a computer using the Statistical Package for Social Science (SPSS) program for statistical analysis (version 25). Data were entered as numerical or categorical as appropriate. The Kolmogorov-Smirnov test of normality of the distribution of the variables was not statistically significant; thus, parametric statistics were adopted.^21^ Data were described using the minimum, maximum, mean, standard deviation, standard error of the mean, 95 % Confidence Interval of the mean, and the 25th to 75th percentiles. Comparisons were performed between two independent normally distributed variables using an independent (Student) *t*-test. The alpha level was set at 5 % with a significance level of 95 %.

## 3 Results

**Fig. 3:**
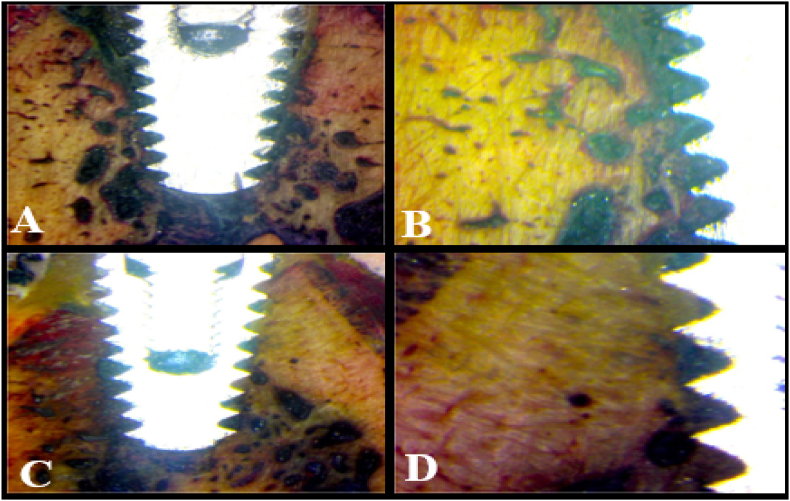
Histological section stained by Stevenes blue and Van Gieson picrofuchsin showing complete adequate bone osteointegration around compromised implant after 8 weeks healing time (Ax100, Bx200). The histological section shows complete osteointegration around control implant after 8 weeks healing time (Cx100, Dx200). (For interpretation of the references to colour in this figure legend, the reader is referred to the Web version of this article.)

All wounds healed uneventfully without any complications. X-ray images revealed successful osteointegration and complete healing of the larger osteotomy site after eight weeks of healing, as evidenced by the presence of high-density bone around the inserted implants (Fig. 3). All inserted implants succeeded in 50 N cm RTT except for one implant in the control group, which recorded 43 N cm on reverse rotation.

**Fig. 4:**
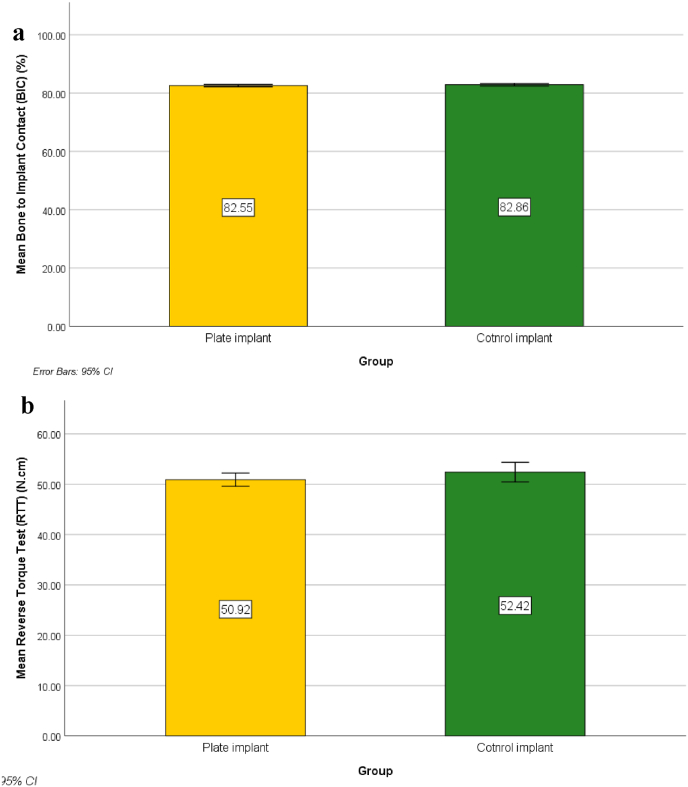
Simple Bar of Mean Reverse Torque Test (RTT) (N.cm) (±95 % CI) by Group(a). Simple Bar of Mean Bone to implant Contact (BIC) (%) (±95 % CI) by Group (b).

Statistical analysis revealed that there was no statistically significant difference in BIC (t = 1.155, p = .260) or RTT (t = 1.408, p = .173), as both implants had almost identical bone-to-implant contact ratio of 82.55 ± 0.61 and 82.86 ± 0.69, respectively (Table 1) (Fig. 4). Histological examination of the prepared hard tissue sections revealed the deposition of high-density bone around both implants and adequate tissue remodeling, as evidenced by the formation of homogenous bone cell lacunae and vascularization of the surrounding bone (Fig. 3).

**Table 1:** Bone to Implant Contact (BIC) and Reverse Torque Test (RTT) measurements (mean ± standard deviations).

	Groups		Test of significance *p*
	Plate implant (n = 12)	Control implant (n = 12)	
**Bone to Implant Contact (BIC) (%)**			
- Min. – Max.	81.40–83.40	81.40–83.80	
- Mean ± SD.	82.55 ± 0.61	82.86 ± 0.69	
- SEM	0.18	0.20	t_(df_ _=_ _22)_ = 1.155
− 95 % CI of the mean	82.16–82.94	82.42–83.30	*p* = .260 NS
− 25th percentile-75th percentile	82.10–82.95	82.70–83.30	

**Reverse Torque Test (RTT) (N.cm)**			
- Min. – Max.	48.00–55.00	48.00–57.00	
- Mean ± SD.	50.92 ± 2.07	52.42 ± 3.06	
- SEM	0.60	0.88	t_(df_ _=_ _22)_ = 1.408
− 95 % CI of the mean	49.60–52.23	50.47–54.36	*p* = .173 NS
− 25th percentile-75th percentile	49.50–52.00	50.00–55.00	

∗: Statistically significant (*p* < .05).

## 4 Discussion

In many clinical situations, a dental implantologist is faced with a situation where bone quality and dimensions compromise the initial stability of the inserted dental implant. In such cases, the surgeon tries to gain some stability by engaging 2 or 3 mm from apical or crestal bone and filling the remainder of the defect size with a bone graft material.^22^ Even in such cases, poor initial stability may result in future implant displacement.^8^^,^^22^ A further drawback is the extended healing time which could exceed several months. Micro-movement of compromised implants could lead to fibrous tissue encapsulation, dislodgment into vital areas as maxillary sinuses or sublingual region, and loss of surrounding bone graft material.^23^

In this study, a microplate fixation plate was used to secure a totally loose dental implant which did not engage any boney wall of the prepared osteotomy. In this extreme condition, the implant was entirely dependent on the assisted initial stability offered by the fixation microplate. The gap between the implant surface and the prepared osteotomy (2 mm in diameter) was left to heal without augmentation with bone graft. This contrasts with a previous study,^24^ in which a mineral-organic adhesive have been used to stabilize an implant put in a region lacking primary stability with engagement of 2 mm apical to the prepared osteotomy. The results with this material were substantially better than those with a bovine bone graft material, which did not stabilize the implant and showed significantly less bone-to-implant contact throughout a 4-month healing period.

The reverse torque test indicated that the compromised implant was able to achieve maximum stability after 8 weeks of healing which was also confirmed analyzing the provided x-rays where the entire defect size was repaired using remodeling of the initially formed blood clot, Fig. 2. Assisted implant fixation resulted in successful osteointegration which reveals the marvelous capacity for bone regeneration without the need for additional tissue guidance or protection. Indeed, the defect size between the compromised implant and the surrounding bone was relatively small to be easily filled with bone, the idea that the compromised implant did not have any contact with the surrounding bone reveals that Assisted implant stabilization was successful in providing sufficient stability during early healing phase. The proposed hypothesis was thus accepted, similar to previous findings for the implants which lacked initial stability.^25^

A prior study reported by Sivolella et al.,^26^ reported osteogenesis on the surface of two loosely placed implants inserted into recipient sites, leaving a 0.7 mm (small) and 1.2 mm (large) circumferential and periapical prepared defect, respectively. While larger defects required several jumps from the woven bone to span the gap, imperfections less than 1 mm gave the opportunity for the newly produced bone to traverse the gap by a single jump.^27^ However, a very low degree of osseointegration was reported. This contradicts our findings because in our study, the gap was 2 mm and osseointegration was successfully achieved. In situations where surrounding teeth are present, or in cases requiring insertion of more than one implant, customized microplate could be used to properly engage the available bone.^28^ Moreover, custom made bands and wires could be adopted to perform the same function.

Examination of the prepared histological sections revealed direct bone implant contact covering the majority of implant surface both the two inserted implants. Dense and vascularized bone formation^29^ in direct contact with implant surface indicated proper healing of the larger osteotomy site prepared around the compromised implant, Fig. 3. Assisted implant stabilization could be performed using guided micro-plate design for complicated cases or those with multiple compromised implants. This is in the agreement with previous study^25^ that revealed the ability of a mineral-organic adhesive to stabilized an implant put in a region lacking primary stability, allow native bone to replace the implant over time without losing structural support, and preserve the crestal bone to the top of the implant.

In cases of one or two wall defect, a bone graft protected by a guidance membrane is the classical approach for insertion of a dental implant in such a compromised osteotomy defect. Incorporation of digital dentistry in the field of design and manufacturing, assisted microplates could be incorporated as a separate component in surgical guides, facilitating insertion and stabilization of several implants inserted in the same jaw.

## 5 Conclusions

Within the limitations of this study, implants inserted in large boney defects lack sufficient primary stability and thus require a costly and lengthy grafting procedure. Modified fixation microplates are easily used to improve primary stability of these implants.

## Patient's/Guardian's consent

There is no patient in our study.

## Clinical Recommendations

Customized fixation microplates could be designed and integrated in digitally made surgical guide which would directly enhance accuracy of this procedure.

## Ethics approval and consent to participate

The animal experiment has been approved by the ethics committee of Tanta University (No. R-OMPDR-6-23-1)

## Consent for publication

Not applicable.

## Availability of data and materials

The data that support the findings of this study are available from the corresponding author upon reasonable request.

## Declaration of competing interest

The authors declare that they have no conflict of interests.

## Abbreviations

BIC: Bone implant contact; RTT: Reverse torque test; *n*: Number of patients; Min-Max: Minimum – Maximum; S.D.: standard deviation; SEM: standard error of the mean; CI: Confidence interval; df: degree of freedom; t: independent sample *t*-test.
